# A behavioural correlate of the synaptic eligibility trace in the nucleus accumbens

**DOI:** 10.1038/s41598-022-05637-6

**Published:** 2022-02-04

**Authors:** Kenji Yamaguchi, Yoshitomo Maeda, Takeshi Sawada, Yusuke Iino, Mio Tajiri, Ryosuke Nakazato, Shin Ishii, Haruo Kasai, Sho Yagishita

**Affiliations:** 1 Laboratory of Structural Physiology, Center for Disease Biology and Integrative Medicine, Faculty of Medicine, Faculty of Medicine Bldg, The University of Tokyo, 1 #NC207, 7-3-1 Hongo, Bunkyo-ku, Tokyo, 113-0033 Japan; 2 International Research Center for Neurointelligence (WPI-IRCN), UTIAS, The University of Tokyo, Bunkyo-ku, Tokyo, Japan; 3 Graduate School of Informatics, Kyoto University, Yoshida-Honmachi, Kyoto, Japan; 4 Present Address: Department of Psychology, Waseda University, Shinjuku-ku, Tokyo, Japan

**Keywords:** Learning and memory, Reward, Learning algorithms

## Abstract

Reward reinforces the association between a preceding sensorimotor event and its outcome. Reinforcement learning (RL) theory and recent brain slice studies explain the delayed reward action such that synaptic activities triggered by sensorimotor events leave a synaptic eligibility trace for 1 s. The trace produces a sensitive period for reward-related dopamine to induce synaptic plasticity in the nucleus accumbens (NAc). However, the contribution of the synaptic eligibility trace to behaviour remains unclear. Here we examined a reward-sensitive period to brief pure tones with an accurate measurement of an effective timing of water reward in head-fixed Pavlovian conditioning, which depended on the plasticity-related signaling in the NAc. We found that the reward-sensitive period was within 1 s after the pure tone presentation and optogenetically-induced presynaptic activities at the NAc, showing that the short reward-sensitive period was in conformity with the synaptic eligibility trace in the NAc. These findings support the application of the synaptic eligibility trace to construct biologically plausible RL models.

## Introduction

Animal behaviours are effectively reinforced when a reward follows a preceding sensorimotor event typically ranging 1–60 s in the conditioning tasks. The time window varies depending on several factors, including type of reinforced behaviour; for example, appetitive licking or lever press typically allow reward delays of 1–3 s^1,2^, whereas approaching behaviour allows delays of 10–60 s^3–6^. To enable such learning, mechanisms are required to associate two temporally separated sensorimotor and reward events flexibly. Reinforcement learning (RL) theory explains that each sensorimotor event evokes an eligibility trace during which a reward can effectively reinforce preceding events^7–10^. Theoretically, the trace can be built up by sequential sensorimotor events occurring during reward learning to yield an accumulating eligibility trace^11^, allowing animals to learn from rewards with diverse delays. Although recent studies have attempted to address neuronal substrates for eligibility traces during reward learning guided by complex sequential sensorimotor events^12–14^, the reward-sensitive periods to a simple sensory input that can closely reflect an eligibility trace before building up remains elusive.

Neuronal substrates for an eligibility trace of reward have been studied as dopamine actions on glutamatergic synapses. Upon unexpected rewards, dopamine neurons in the ventral tegmental area (VTA) show a phasic burst firing (~ 0.3 s)^15,16^, which is regarded to represent a reward prediction error signal in the RL theory. Following optogenetic studies supported this idea by showing that the phasic dopamine activity is sufficient and indispensable to establish reward learning^2,17–19^. VTA dopamine neurons send dense projection to the nucleus accumbens (NAc), which also receives glutamatergic inputs from several brain regions such as the amygdala. The amygdala sends sensory information of the CS^20^ and the amygdala to NAc pathway is required for auditory cue-reward association^21,22^. The dopaminergic and glutamatergic inputs signal through dopamine D1 receptors (D1Rs) and *N*-methyl-d-aspartate type glutamate receptors (NMDARs) in the NAc for reward conditioning^23,24^. In slice preparations, D1R, NMDAR, and Ca^2+^/calmodulin–dependent protein kinase II (CaMKII) regulate the enlargement of the dendritic spine, a structural basis for long-term potentiation of the D1R-expressing spiny projection neurons (D1-SPNs)^25^. Of note, pairing of glutamatergic inputs and postsynaptic action potentials shaped the dopamine-sensitive period for plasticity only about 1 s^25–29^.

These lines of evidence suggest that synaptic activities triggered by sensorimotor events leave synaptic eligibility traces for 1 s in the NAc, a time window during which reward-related dopamine could induce plasticity for behavioural learning. This cellular mechanism corresponds to the theoretical model of NeoHebbian three-factor learning rules, which requires a third factor such as dopaminergic inputs as well as Hebbian concurrent presynaptic and postsynaptic activities to update weights of neuronal connections^8^. However, several different neuronal mechanisms may exist in the brain for different types of eligibility traces. For example, outside the NAc, synaptic eligibility traces have been found to have longer time scales of 5 s in the neocortex^30^ and 10 min in the hippocampus^31^. In addition to synaptic eligibility traces, persistent activities that store eligible events in working memory can also associate temporally separated events^32^.

To clarify the contribution of the synaptic eligibility trace in the NAc in vivo, we sought to examine the reward-sensitive period around a short auditory input in a Pavlovian conditioning task with head-restrained mice. The water of reward was directly delivered to the mouth of mice to accurately present the unconditioned stimuli (US) without any delay before consumption. This tone-water-licking task enabled the rapid establishment of conditioning within an hour, in contrast to tasks where licking is reinforced by water (antecedent-licking-water operant conditioning) which requires several days for their acquisition^33^ and involves brain regions such as the prefrontal cortex (PFC)^12,34,35^. We examined the reward-sensitive periods of the conditioned stimuli (CSs) and tested the dependence of the conditioning on the NAc. We further applied optogenetic stimulation of synaptic inputs to NAc to eliminate the possible delay of the sensory stimulus to the NAc.

## Results

### Rapid Pavlovian conditioning with a short CS in head-restrained mice

**Figure 1:**
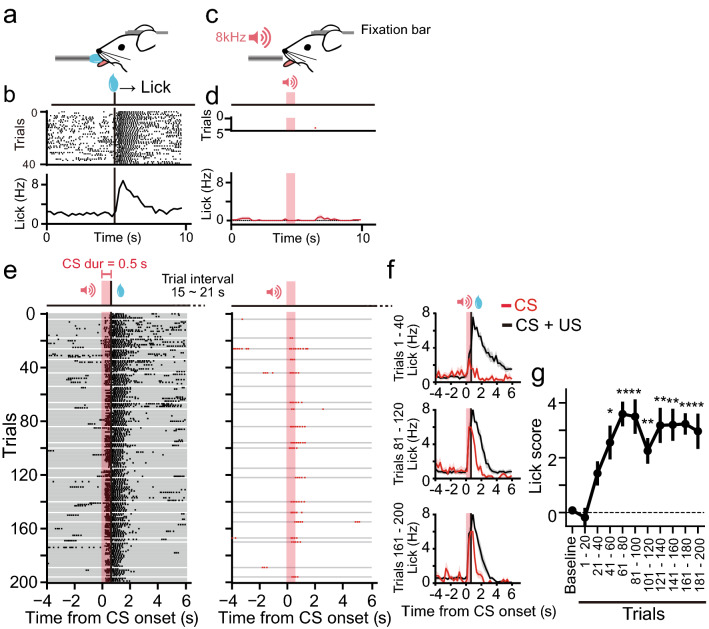
Pavlovian conditioning in head-restrained mice. (**a**) A schematic diagram of the behavioural setup. Mice were head-restrained using a chronically implanted bar. A drop of glucose water was presented as an US to directly touch the mouth of the mouse from a tube that monitored the licking responses. (**b**) Raster plots (upper) and an averaged peristimulus time histogram (PSTH, bottom) for the licking responses to US presentation from a representative mouse. The US was presented 40 times at 10-s intervals. Black vertical bars indicate the onset of US presentation. (**c**) A short tone (8 kHz, 70 dB, 0.5 s) presentation without US. (**d**) Raster plots (upper) and an averaged PSTH (bottom, *n* = 7 mice) for licking responses to CS before conditioning. The baseline measurement consisted of five consecutive trials with CS-only presentation. Red shades indicate the period of CS presentation. (**e**) Raster plots for licking during conditioning with a CS duration of 0.5 s in a representative mouse. The session consisted of 180 trials with a paired presentation of CS and US (left) and pseudo-randomly inserted 20 trials with CS-only presentation (right). “CS dur” indicates the duration of CS presentation. US was presented at the offset of CS. Intervals between CS presentations were pseudo-randomly varied between 15 and 21 s. Each gray line indicates a single trial. (**f**) Averaged PSTHs for the licking responses in the first 20% of the trials (1–40, upper), the third 20% of the trials (81–120, middle), or the last 20% of the trials (161–200, bottom) during CS + US trials (black trace) or CS-only trials (red trace). In the CS-only trials, the average of four trials was included in the indicated periods are shown. The shadows indicate SEM. *n* = 7 mice. (**g**) Lick scores (“Methods”) plotted against time. *P < 0.05, **P < 0.01 (*n* = 7 mice, Kruskal–Wallis test). Error bars indicate SEM.

We used a head-restrained device to deliver a US of water at an arbitrary timing for Pavlovian conditioning. The position of the licking port was set close to the mouth of the mice (Fig. 1a) so that a drop of water would immediately touch the mouse to signify delivery of the US. Thus, licking responses (UCR) were induced just after the presentation of the US (Fig. 1b). Before conditioning, we measured baseline responses to a short, pure tone (8 kHz, 0.5 s) (Fig. 1c), which was subsequently used as the CS, and confirmed that the tone itself did not evoke a licking response (Fig. 1d). For the tone-water-licking conditioning, we presented a CS followed by a US at the CS offset (0.5 s) for 180 trials (Fig. 1e,f). To monitor the formation of the association during conditioning, 20 CS-only trials were pseudo-randomly inserted among the 180 trials with CS–US presentation so that 2 CS-only trials were included in every 20 trials. The learning curve of the conditioning was obtained by plotting the lick scores calculated using the averaged licking frequency for 2 s from the onset of CS, which was subtracted from the lick frequency 2 s before CS (Fig. 1g). The results showed that mice started to predict US arrival at the presentation of the CS after 40 trials of pairing, and learning was saturated after 120 trials (Fig. 1g, Kruskal–Wallis test, χ^2^(10) = 39.8, *P* = 1.8 × 10^−5^; post-hoc Steel’s test: Baseline vs. 1–20, *P* = 0.97; vs. 21–40, *P* = 0.36; vs. 41–60, *P* = 0.014; vs. 61–80, *P* = 0.0065; vs. 81–100, *P* = 0.0065; vs. 101–120, *P* = 0.0064; vs. 121–140, *P* = 0.0064; vs. 141–160, *P* = 0.0065; vs. 161–180, *P* = 0.0065; vs. 181–200, *P* = 0.0065).

Next, we attempted to identify the optimal range of CS duration by altering CS durations (0.2 s, 0.5 s, 1 s, 2 s, 3 s, and 4 s) when USs were applied at the offset of the CSs (Supplementary Fig. S1 online). A CS duration of 0.5 s was associated with a significant increase in the licking response after conditioning (Wilcoxon signed-rank test, Baseline vs*.* Trial 161–200: Z = − 2.37, *P* = 0.016). Although a gradual increase in lick frequency was observed across CS durations of 0.2–3 s, no CS duration other than 0.5 s reached statistical significance (Wilcoxon signed-rank test, Baseline vs*.* Trial 161–200: for 0.2 s, Z = − 1.83, *P* = 0.13; for 1 s, Z = − 2.02, *P* = 0.063; for 2 s, Z = − 1.10, *P* = 0.34; for 3 s, Z = − 1.83, *P* = 0.13; for 4 s, Z = 0.40, *P* = 0.81). Thus we used a tone duration of 0.5 s in the following experiments as a short and optimal CS.

### Reward-sensitive period to brief CS in NAc-dependent Pavlovian conditioning

**Figure 2:**
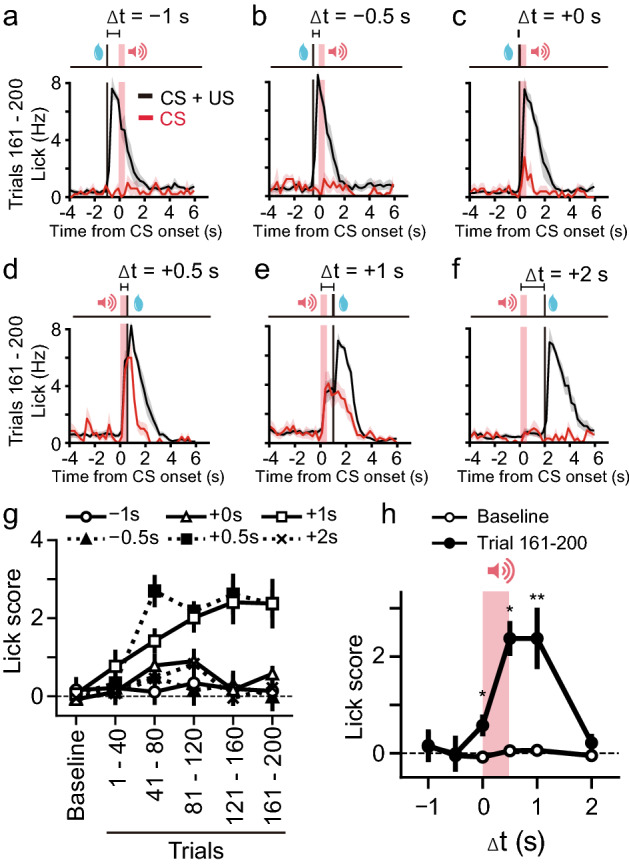
Conditioning with various delays of the US. (**a**–**f**) Averaged PSTHs of the licking responses with delays of − 1 s (**a**, n = 5 mice), − 0.5 s (**b**, n = 5 mice), + 0 s (**c**, n = 5 mice), + 0.5 s (**d**, n = 7 mice), + 1 s (**e**, n = 11 mice), or + 2 s (**f**, n = 7 mice) during trials 161–200. The plot in (**d**) is the same as that at the bottom of Fig. 1f. Red shades indicate the period of CS presentation. (**g**) Peak lick scores plotted against time. Each symbol represents the delay in the US relative to the CS. (**h**) Time windows for US presentation leading to conditioning. The averaged lick scores in the baseline session (white circle) and CS-only trials included during conditioning trials 161–200 (black circle) were plotted against delays between the CS and US. Wilcoxon signed-rank test. *P < 0.05, **P < 0.01.

We then determined the reward-sensitive period to a CS of 0.5 s by presenting US with various delays (Fig. 2a–f). When the US preceded the CS, the CS did not induce licking responses after conditioning (Fig. 2a,b). The mice rapidly predicted the US when the CS preceded the US by no more than 1 s (Fig. 2c–e). However, a CS–US interval of 2-s did not allow the formation of the association (Fig. 2f). The difference in peak frequency between + 0.5 s (Fig. 2d) and + 1 s (Fig. 2e) was consistent with evidence from prior studies showing that frequency of responses to CSs decreases as the CS–US interval gets longer^33^. The lick scores were calculated from the averaged licking frequency for 2 s after CS presentation subtracted from that 2 s before CS presentation to plot a learning curve (Fig. 2g) and time window (Fig. 2h). We found that the reward-sensitive period was only within 1 s after the short tone (Fig. 2h) (Wilcoxon signed-rank test, Baseline vs. Trial 161–200: − 1 s, Z = 0.13, *P* = 0.89; − 0.5 s, Z = 0.67, *P* = 0.5; + 0 s, Z = 2.02, *P* = 0.043; + 0.5 s, Z = 2.36, *P* = 0.017; + 1 s, Z = 2.48, *P* = 0.012; + 2 s, Z = 1.18, *P* = 0.23).

### NAc-dependence of the conditioning

**Figure 3:**
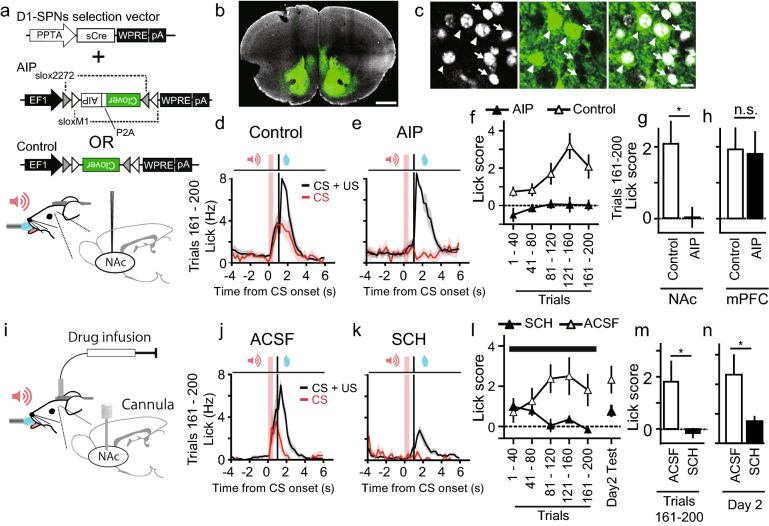
The effect of a D1 receptor blocker and CaMKII inhibitory peptide (AIP) in the NAc on conditioning. (**a**) Viral constructs and schematics of injection. (**b**,**c**) Confocal images of clover fluorescence (green) and DAPI (white) from a coronal slice, including the NAc. Slices were counter-stained with DAPI, and 35.8% (72/201) of cells were positive for AIP. Arrowheads indicate AIP-positive cells, and arrows indicate negative cells. Scale bars indicate 1 mm (**b**) and 20 μm (**c**). (**d**,**e**) Averaged PSTHs of the licking responses in CS + US (black) and CS (red) conditionings from mice injected with control (**d**, *n* = 7 mice) or AIP (**e**, *n* = 6 mice). (**f**) The peak lick scores are plotted against time for the conditions indicated. (**g**,**h**) Average lick scores from eight trials during trials 161–200 from mice with injections in the NAc or injections in the mPFC (this figure, Supplementary Fig. S2 online) were plotted for each condition. *P < 0.05, two-sided Mann–Whitney *U* test. (**i**) A schematic diagram for drug infusion into the NAc. (**j**,**k**) Averaged PSTHs of the licking responses in trials 161–200 without (**j**, ACSF, *n* = 5 mice) or with (**k**, SCH23390, *n* = 5 mice) the injection of a D1 blocker during conditioning. (**l**) The lick scores were plotted against time for the two conditions indicated. Drug infusion was started 30 min before the behavioral experiments and was continued during conditioning as indicated by the black horizontal bar. (**m,n**) The effect of a dopamine D1 receptor blocker, SCH23390, on conditioning. Averaged lick scores of CS-only trials during trials 161–200 (**m**) or day 2 (**n**) were plotted. *P < 0.05, two-sided Mann–Whitney *U* test, *n* = 5 mice for SCH23390, *n* = 5 mice for ACSF.

We tested whether the molecular signaling required for plasticity in the NAc is indispensable for the rapidly forming conditioning. We first examined CaMKII signaling by an autocamtide 2-related inhibitory peptide (AIP), a peptide that inhibits CaMKII activity^36^, with which we previously showed that AIP expression in the SPNs prevented plasticity and learning^37^. Then, Adeno-associated virus (AAV) vector with a PPTA promoter for D1-SPNs^25^ (Fig. 3a) was injected bilaterally into the NAc, and the extent of the expression was monitored by a green fluorescent protein that was co-expressed with AIP using a P2A cleavage site (Fig. 3b,c). We tested the behavioural effects of AIP expression in the NAc and found that the AIP expression in the NAc abolished learning (Fig. 3d–g) (two-sided Mann–Whitney *U* test, *U* = 3, *P* = 0.01). In contrast, expression of AIP in the prefrontal cortex (PFC) under a CaMKII promoter did not affect conditioning (Fig. 3h, Supplementary Fig. S2 online) (two-sided Mann–Whitney *U* test, *U* = 14, *P* = 0.56). These results indicated that the current rapid conditioning task preferentially relied on the NAc molecular signaling related to plasticity, unlike other reward conditioning that involves the PFC^12,34,35^, which may have longer eligibility trace^30^.

Next, we injected a dopamine D1R antagonist (SCH23390) in the bilateral NAc during conditioning (Fig. 3i). A D1R antagonist blocked the conditioning when the CRs were measured at the end of conditioning (Fig. 3j–m) (two-sided Mann–Whitney *U* test, *U* = 3, *P* = 0.044). The D1R antagonist also partially inhibited US responses, suggesting that D1R inhibition also affected motor components. Furthermore, CRs on the following day where no drug was present were also inhibited in mice with the D1R antagonist (Fig. 3n) (two-sided Mann–Whitney *U* test, *U* = 3, *P* = 0.047), supporting that the D1R antagonist blocked conditioning.

### Reward-sensitive period to optogenetic stimulation of the synaptic input to the NAc

**Figure 4:**
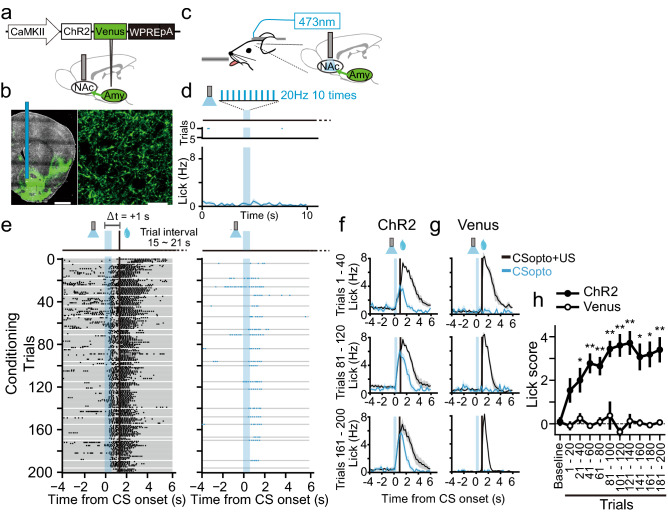
Pavlovian conditioning with CSopto. (**a**) A viral construct and schematic of the AAV injection and optical fibre implantation. ChR2-expressing AAV was injected into the left basolateral amygdala (BLA). An optical cannula (200 μm core) was placed into the left NAc. (**b**) Macroscopic (left) and microscopic (right) confocal images of the green fluorescence of Venus fused with ChR2 in the NAc. A blue vertical bar indicates the tract of the inserted optical cannula. Scale bars indicate 1 mm (left) and 20 μm (right). (**c**) Schematic of the behavioural setup. The optical cannula was connected to a laser (473 nm) by a patch cable. (**d**) Representative licking responses before conditioning in response to ChR2 stimulation (CSopto, 20 Hz, ten times, 5 ms pulse width). Raster plots indicating licking responses from a representative mouse and PSTHs indicating averaged responses over ten mice. Blue shades indicate the period of CSopto presentation. (**e**) Representative licking responses during CSopto conditioning with Δt = + 1 s. The conditioning paradigm was the same as Fig. 1 except that the tone was replaced with CSopto, and the delay was 1 s. Raster plots indicating licking responses from CS + US trials (left) or CS-only trials (right). Each gray bar indicates a single trial. Black vertical bars indicate the onset of US presentation. (**f**,**g**) Averaged PSTHs for the licking responses in the first 20% of the trials (1–40, upper), the third 20% of the trials (81–120, middle), or the last 20% of the trials (161–200, bottom) for each of the CS + US trials (black trace) or the CS-only trials (blue trace) from mice injected with ChR2 (**f**, *n* = 10) or Venus (**g**, *n* = 4). Shadows indicate SEM. (**h**) Lick scores (“Methods”) plotted against time course for the ChR2 mice (*n* = 7) and Venus mice (*n* = 4). Kruskal–Wallis test. *P < 0.05, **P < 0.01. Error bars indicate SEM.

Although we found the 1 s of reward-sensitive period in the NAc-dependent conditioning task, it is still possible that the observed window was formed upstream of the NAc and the NAc mechanism was far shorter. To exclude this possibility, we applied optogenetics to stimulate glutamatergic inputs to the NAc directly. Previous studies showed that the basolateral amygdala (BLA) to NAc pathway represents CS information^20–22^, and also reinforces behaviours^22^. We hypothesized that weak optogenetic stimulation of this pathway acts as a CS while strong stimulation acts as a reinforcer. The ChR2-expressing AAV vector was injected into the left amygdala, and an optical fibre was placed in the ipsilateral NAc (Fig. 4a,b). First, we replicated reinforcement effects of the BLA to NAc pathway (Supplementary Fig. S3 online) by stimulating axonal fibres (457 nm, 5 ms, 20 Hz, ten times) at high (> 5 mW) laser power (Supplementary Fig. S3 online) (Kruskal–Wallis test, χ^2^(3) = 19.1, *P* = 0.0003; post-hoc Steel’s test: laser on at low power vs. laser off, *P* = 0.87, laser on at high power vs. laser off, *P* = 0.0036, laser on at low power vs. laser on at high power, *P* = 0.0019). In contrast, subthreshold low laser powers (< 3 mW) did not reinforce this behaviour (laser on at low power vs. laser off at low power, *P* = 0.87) (Supplementary Fig. S3 online).

We then tested whether this weak stimulation of synaptic inputs (optogenetic conditioned stimulus, CSopto) could be associated with the US. In head-fixed mice, blue light stimulation (20 Hz, 0.5 s, 5 ms pulse) of CSopto alone in the NAc did not cause the licking response (Fig. 4c,d). When CSopto was paired with a US of water (Fig. 4e,f), the mice started to show anticipatory licking to CSopto within 40 trials (Fig. 4e,f,h, Kruskal–Wallis test, χ^2^(10) = 32.3, *P* = 0.00035; post-hoc Steel’s test: Baseline vs. 1–20, *P* = 0.058; vs. 21–40, *P* = 0.048; vs. 41–60, *P* = 0.0013; vs. 61–80, *P* = 0.008; vs. 81–100, *P* = 0.001; vs. 101–120, *P* = 0.0013; vs. 121–140, *P* = 0.0033; vs. 141–160, *P* = 0.022; vs. 161–180, *P* = 0.022; vs. 181–200, *P* = 0.0043). In contrast, mice injected with a Venus vector without ChR2 did not form an association (Fig. 4g,h) (Kruskal–Wallis test, χ^2^(10) = 6.52, *P* = 0.76), indicating that mice did not respond to optical stimulation itself as a CS but the conditioning relied on optically induced synaptic activation. Moreover, CSopto conditioning was dependent on the D1R, which was tested using a within-subject design to functionally confirm virus injection and fibre placement for ChR2 excitation (Supplementary Fig. S4 online, two-sided Mann–Whitney *U* test, *U* = 3, *P* = 0.018).

**Figure 5:**
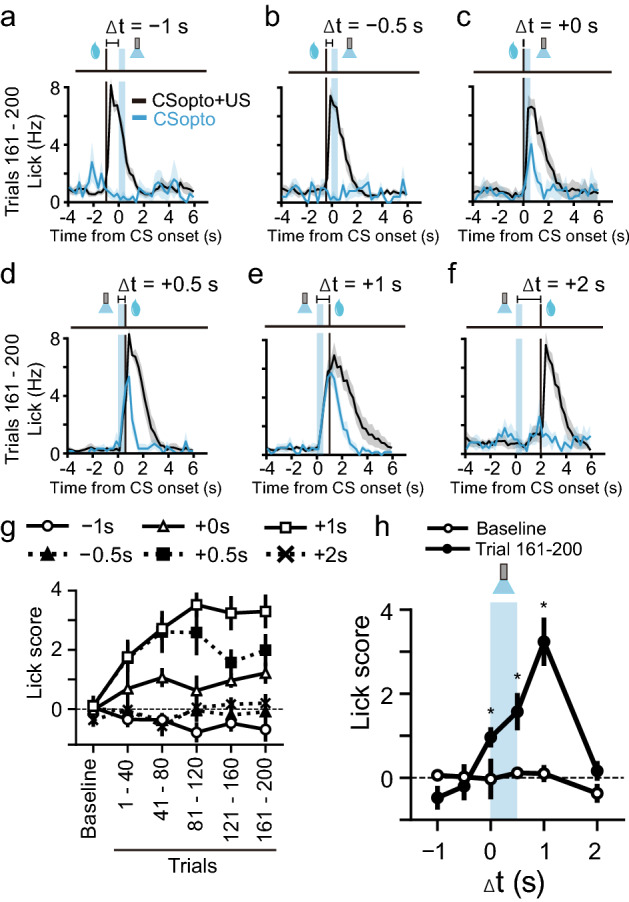
Various delays in US for CSopto conditioning. (**a**–**f**) Averaged PSTHs of the licking responses in conditioning with delays of Δt = − 1 s (**a**, *n* = 5 mice), Δt = − 0.5 s (**b**, *n* = 5 mice), Δt = + 0 s (**c**, *n* = 5 mice), Δt = + 0.5 s (**d**, *n* = 6 mice), Δt = + 1 s (**e**, *n* = 10 mice), and Δt = + 2 s (**f**, *n* = 5 mice) during 161–200 trials. (**e**) is the same plot as the bottom trace of Fig. 4f. (**g**) Lick scores plotted against time course for each condition. (**h**) Time window for conditioning. Lick scores in test trials or eight trials during trials 161–200 were plotted against delays between the CS and US. Wilcoxon signed-rank test. *P < 0.05.

Finally, we examined reward-sensitive periods for the CSopto (20 Hz, 0.5 s) (Fig. 5). The time window of conditioning by the CSopto was within 1 s after the onset of CSopto (Fig. 5h) (Wilcoxon signed-rank test, Baseline vs. Trial 161–200: − 1 s, Z = 1.75, *P* = 0.079; − 0.5 s, Z = 0.94, *P* = 0.34; + 0 s, Z = 2.02, *P* = 0.043; + 0.5 s, Z = 1.99, *P* = 0.046; + 1 s, Z = 2.59, *P* = 0.0093; + 2 s, Z = 1.21, *P* = 0.22), similar to the natural tone (Fig. 2h). For the negative conditions (− 1 s, − 0.5 s, and 2 s), we confirmed successful conditioning with 1 s delay on the next day (Supplementary Fig. S5 online), indicating that the negative results were not due to inappropriate virus injection or optical fibre placement.

## Discussion

We demonstrated that the reward-sensitive period was 1 s after the brief CS, which was similar even with the optogenetic stimulation of glutamatergic inputs in the NAc with a Pavlovian conditioning task in head-restrained mice. The period was in good agreement with the temporal profile of synaptic eligibility trace in the NAc. Thus, our data provide a behavioural line of evidence to apply the timing of the synaptic eligibility traces to construct RL models.

At the molecular level, the time window of 1 s suggests that the temporal scale is mainly determined by a signaling pathway involving D1R, Ca^2+^ priming of adenylate cyclase (AC), protein kinase A (PKA), and CaMKII^25,28,29^. Previous studies have shown that distal dendrites exhibit high phosphodiesterase activity that suppresses the increase in cAMP concentration even in the presence of reward-related phasic dopamine input which activates the cAMP production pathway of D1R-G_s/olf_-AC^25,28^. When postsynaptic action potentials cause Ca^2+^ influx, Ca^2+^-sensitive AC is primed for 1 s so that dopamine can outcompete phosphodiesterase activity to allow cAMP to increase, which in turn activates PKA. PKA then disinhibits CaMKII specifically at the spine, which receives presynaptic glutamatergic inputs concurrently with postsynaptic activity^25,28^. This time window of 1 s is longer than another major time window determined by NMDA receptors that detect concurrent presynaptic and postsynaptic activities for plasticity at ~ 50 ms^38^. This indicates that the synaptic eligibility trace mechanism effectively prolongs the duration of reward detection but compromises precision in detection of temporal contiguity. Interestingly, similar molecular timing mechanisms associated with Ca^2+^-sensitive AC have been found in Aplysia^39,40^ and in insects^41–43^, suggesting that the neuronal mechanism involving Ca^2+^-sensitive AC may resolve the tradeoff between the sensitivity and precision.

The short NAc eligibility trace predicts that NAc plasticity becomes predominant when reward immediately follows preceding sensory events. For example, the visual and olfactory cues of foods are usually present immediately before tasting. The palatable reward of foods thus can strongly reinforce sensory cues by the synaptic eligibility trace in the NAc so that only the sensory cue can subsequently activate the NAc. The NAc strongly reacts to sensory cues of foods both in human^44,45^ and rodents^46^. Rapid action of addictive substances taken by inhalation or injections would explain the NAc reactions to predictive cues^47^. Thus, the short synaptic eligibility trace may explain why the NAc activities react to the sensory information of reward itself.

The three factors of the presynaptic input, postsynaptic action potentials of SPNs, and dopamine may contain specific information for learning, assuming the involvement of synaptic eligibility trace. Several lines of behavioural evidence support the idea that the presynaptic input represents the CS^20–22^ and dopamine activity represents a reward prediction error^15–19^. In contrast, the exact information represented by postsynaptic action potentials has not been well clarified. We argue two possible models here. One model is that the postsynaptic action potentials cause licking behaviours by activating downstream brainstem nuclei^48,49^. Consistent with this idea, we showed that CSopto induced a transient, rhythmic licking movement, supporting the existence of a licking pathway downstream of the NAc. Spontaneous licking occurred even before establishment of learning (baseline licking in Fig. 1f) once after water presentation (baseline licking in Fig. 1b vs. d), suggesting that licking-related postsynaptic activities during the CS period may fire together with CS-related presynaptic inputs to generate a synaptic eligibility trace so that subsequent dopamine inputs can cause plasticity for autoshaping of conditioning. Instead, a Pavlovian association model requires licking-related postsynaptic activities during US periods to be associated with preceding CS-related presynaptic activities. In this scenario, CS-induced presynaptic activities and US-induced postsynaptic activities are separated by intervals up to 1 s which cannot cause plasticity given the known synaptic mechanisms in the NAc but can do so in the hippocampus^50^. The other model is that CS-related presynaptic inputs cause dendritic spikes instead of action potentials to induce plasticity^51^ when subsequent dopamine inputs arrive; once synaptic weights have been enhanced by this plasticity, CS-related presynaptic activity can trigger action potentials. A limitation of this model is that it cannot explain why particular behaviours, licking responses in our study, are selectively reinforced during conditioing. The actual circuit model needs to be clarified in future studies by visualization of learning-related circuits and timing-specific neuronal manipulation of relevant neural circuits.

Even without eligibility traces, a temporal-difference (TD) algorithm provides a model for explaining associations between two temporally separated events. In the TD model, time is represented in a discrete state and the reward value is initially associated with the state at the timing of reward. Then, after learning has proceeded through multiple trials, the value gradually shifts back to the onset of the CS^15^. This model can explain associations between two temporally separated events at any interval given a sufficient number of trials, which is inconsistent with our observation of the time window. It is still possible, however, that a gradual backward shift of licking occurred in our study, a pattern which is predicted by TD learning theory. Although we observed no apparent shifting of licking responses using a short auditory CS (Fig. 1), a definitive analysis was difficult because of ambiguous onset of licking due to baseline responses measured during the early period of conditioning. As shown in a human study, development of one-shot learning is needed to exclude involvements of the TD learning pattern^52^. In one previous study with rats, it was found that CS-induced dopamine responses did not follow the TD learning pattern but instead exhibited a CS-induced response at the onset of the CS, a pattern consistent with learning models involving eligibility traces in conditioning with a CS–US interval of 1 s^53^. Interestingly, in a recent study with mice in which an olfactory CS and CS–US intervals of 3 s were used, the investigators observed gradual shifts toward the onset of CSs over multiple trials^54^, suggesting that TD mechanisms also play a role in learning but with longer intervals than the synaptic eligibility trace.

Ethologically relevant behaviours require longer reward time windows than the synaptic eligibility traces. Working memory-like mechanisms may send persistent inputs to the NAc^32^, which may activate the synaptic eligibility trace even after the cessation of external sensory inputs. Second-order conditioning, where reward predicting CS becomes a reinforcer for other preceding events, also allows learning from longer reward delays^15,54,55^. Synaptic mechanisms with more prolonged eligibility traces outside the NAc^30,31^ can play direct roles in complex reward learning^12,34,56^. How the NAc and additional brain mechanisms interplay during complex reward learning will be a future research focus.

In conclusion, we identified that the reward-sensitive period was 1 s in the NAc-dependent rapid conditioning task, which is in close agreement with the dopamine-sensitive period for synaptic plasticity in the NAc. Such biologically defined temporal constraints may help to understand and construct biologically plausible RL models.

## Methods

### Adeno-associated virus (AAV) preparation

We cloned the following AAV-expression plasmids: pAAV-CaMKII(0.3)-hChR2(H134R)-Venus, pAAV-CaMKII(0.3)-Venus, pAAV-PPTA-sCre, pAAV-sDIO(M1)-Clover-P2A-AIP, pAAV-sDIO(M1)-Clover, pAAV-CaMKII(0.3)-mCherry-P2A-AIP and pAAV-CaMKII(0.3)-mCherry. The PPTA promoter, a D1-SPN specific promoter, was cloned from the mouse as described previously^25,57^. Autocamtide 2-related inhibitory peptide (AIP), a CaMKII inhibitory peptide, and self-cleaving 2A peptide of porcine teschovirus-1 (P2A) were fused with clover and cloned in a sCre dependent double inverted ORF expression vector designed using sloxP and sloxP (M1). The original plasmid containing hChR2(H134R) was a kind gift from Dr. Deisseroth, and sCre was purchased from Kazusa DNA Research Institute (Japan)^58^. AAV vectors were produced, and their titers were measured as described previously^59^. Briefly, plasmids for the AAV vector, pHelper (Stratagene), and RepCap5 (Applied Viromics) were transfected to HEK293 cells (AAV293, Stratagene). After 3 days of incubation, the cells were collected and purified twice using iodixanol. The titers for AAV were estimated using a quantitative polymerase chain reaction.

### Animals and surgery

Wild type or DAT-IRES-Cre (B6.SJL-Slc6a3tm1.1(cre)Bkmn/J, The Jackson Laboratory) male B6J mice aged 2–4 months old were used. These mice were housed on a 12-h light/12-h dark cycle. A custom-made titanium plate was attached to the head using dental cement. For AIP experiments in the NAc, a total of 1.5 μl of the AAV mixture of PPTA-sCre (5 × 10^11^ GC/ml) with either EF1-sDIO(M1)-Clover-P2A-AIP (2 × 10^13^ GC/ml) or EF1-sDIO(M1)-Clover (1 × 10^13^ GC/ml) were bilaterally injected (AP + 1.3 mm, ML ± 1.0 mm, DV + 4.5 mm) through a glass pipette. For AIP experiments in the medial prefrontal cortex (mPFC), 1.5 μl of CaMKII (0.3)-mCherry-P2A-AIP (2 × 10^13^ GC/ml) or CaMKII(0.3)-mCherry (2 × 10^13^ GC/ml) were bilaterally injected (AP + 1.8 mm, ML ±  0.3 mm, DV + 2.5 mm). The infusion rate was controlled using a syringe pump set at 0.05–0.1 µl/min. For the ChR2 experiments, 1 µl of CaMKII(0.3)-ChR2-Venus (2–3 × 10^13^ GC/ml) or CaMKII(0.3)-Venus (2–3 × 10^13^ GC/ml) was injected into the left basolateral amygdala (AP − 1.6 mm, ML − 3.3 mm, DV + 4.7 mm). After injection, an optical fibre cannula (200 μm core, 5.0 mm in length, Thorlabs, CFML12U) was inserted into the left NAc (AP + 1.4 mm, ML − 0.75 mm, DV + 4.1 mm). For the drug infusion experiments, a 5.0 mm double guide cannula (26-gauge, 1.5 mm apart from each cannula, Plastic One) were implanted bilaterally into the NAc (AP + 1.3 mm, ML ± 0.75 mm, DV + 4.2 mm). The experimental protocol was approved by the Animal Experimental Committee of the Faculty of Medicine, The University of Tokyo. All methods were carried out in accordance with the institutional guidelines and in compliance with the ARRIVE guidelines. Researchers were not blined to the group allocation.

### Behavioural experiments

Mice were allowed 4 days for recovery after head plate installation in experiments without virus injections and 3 weeks for recovery in experiments with virus injections. Mice were then habituated for 3 days to the experimental setup without head fixation, and water restricted such that body weight was maintained at no less than 80% of the baseline weight. On the day of the experiment, the mice were head-fixed, and the licking responses to tone presentation (8 kHz, 70 dB) used as CS were monitored for five trials (day 1, baseline session). For the US, a drop of 5% glucose water (2 μl) was presented through the tip of a lick port controlled by a syringe pump. The position of the lick port was set such that the drop of water contacted the mouth of the mice to induce licking without any training. The conditioning session consisted of 180 trials with the presentation of CS–US pairs and 20 trials with the presentation of CS only. For the time window experiment, each mouse was assigned to one of the CS–US delays of − 1 s, − 0.5 s, 0 s, + 0.5 s, 1 s, or 2 s with CS duration of 0.5 s. For the CS duration experiment, each mouse was assigned to one of the CS duration of 0.2 s, 1 s, 2 s, 3 s, or 4 s. The data from the mice assigned to CS–US delays of + 0.5 s were also used as that of the CS duration of 0.5 s. The intervals between the trials were randomized with a uniform distribution between 15 and 21 s, with a mean of 18 s. To monitor learning during conditioning, CS-only trials were pseudo-randomly inserted so that two trials with CS only were included in every of 18 CS–US trials to record conditioned reflexes (CRs) without US. The licking responses were electrically measured. The control of the stimulus presentations and the recording of the licking responses were performed with custom software written in LabView (National Instruments).

For experiments with ChR2 stimulation, a fibre cannula was connected to a blue laser (473 nm, Thorlabs). For the operant conditioning session^22^ shown in Supplementary Fig. S3, conditions with laser on and off were alternately repeated twice. In the laser-on condition, axonal fibres were stimulated (5 ms pulse, ten times in 20 Hz) 100 ms after the detection of a licking event while no stimulation was made in the laser off condition. After the stimulation, we inserted a 500-ms refractory period for stimulation, even though the sensor detected licking. The number of licking responses was counted for 190 s. To initiate licking, the lick port delivered a drop of water once 10 s before recording. The session was repeated with increasing laser power from 1, 2, 3, 5, 7.5 to 15 mW (200 μm core fibre) or until the mice lick counts during the laser-on period were 20 times greater than those during the laser off period. For Pavlovian conditioning with ChR2, 20-Hz laser stimulation (5 ms pulse, 1 or 2 mW) given 10 times (CSopto) was substituted for the CS tone.

For the drug infusion experiment, SCH23390 (400 μM, Abcam) dissolved in ACSF (125 mM NaCl, 2.5 mM KCl, 2 mM CaCl_2_, 1 mM MgCl_2_, 1.25 mM NaH_2_PO_4_, 26 mM NaHCO_3_, and 20 mM glucose) or ACSF for controls was infused at the rate of 16.66 nl/min by a syringe pump (Legato111, KD scientific) 30 min before the experiments. The infusion was continued during the conditioning at the rate of 14.9 nl/min. For pharmacological experiments during CSopto conditioning, SCH23390 or saline were intraperitoneally injected 30 min before the conditioning experiments. Doses of 0.25 and 0.5 mg/kg were tested. As the results were similar between the doses, the data were pooled in the analysis.

### Histological analysis

For the AIP experiments, the mice were subjected to histological analysis to confirm AIP expression in the NAc. After the behavioural experiments, the mice were transcardially perfused with 4% paraformaldehyde and decapitated. Coronal slices of 50-μm thickness were obtained. Clover fluorescent was obtained using stereoscopic microscopy (Leica M165-FC), and images were captured with a CMOS camera (Hamamatsu photonics ORCA R2). AIP expression was considered sufficient if it was expressed bilaterally, including more than 3/4 of the anterior part of the anterior commissure, a NAc surrounding structure. Out of the 18 NAc-injected mice, five failed to satisfy this criterion (one did not exhibit expression at all, three exhibited unilateral expression only, and one exhibited expression only in the medial half of the NAc) and were therefore excluded from behavioural analyses. For some slices, detailed fluorescence images were obtained using confocal microscopy (Leica, SP5) of the preparations, which were counter-stained using DAPI.

### Data analysis

For the analysis of the CS-induced licking responses (CRs), we calculated the lick score in the CS-only trials as [average licking frequency (Hz) during 2 s after CS presentation] − [average licking frequency during 2 s before CS presentation]. Kruskal–Wallis test followed by Steel test or *t* test were adapted for statistical tests with a threshold of *P* < 0.05. Wilcoxon rank-sum test, Mann–Whitney test. Data analyses were performed using Excel (Microsoft) and Excel Statistics (SSRI). Data are presented as mean ± SEM.

## Supplementary Information

Supplementary Figures.

## Data Availability

All data are available from the corresponding author upon reasonable request.

## References

[1] Black J, Belluzzi JD, Stein L (1985). Reinforcement delay of one second severely impairs acquisition of brain self-stimulation. Brain Res..

[2] Lee K (2020). Temporally restricted dopaminergic control of reward-conditioned movements. Nat. Neurosci..

[3] Holland PC (1980). CS–US interval as a determinant of the form of Pavlovian appetitive conditioned-responses. J. Exp. Psychol. Anim. Behav. Process..

[4] Akins CK, Domjan M (1996). The topography of sexually conditioned behaviour: Effects of a trace interval. Q. J. Exp. Psychol. B.

[5] Akins CK, Domjan M, Gutiérrez G (1994). Topography of sexually conditioned behavior in male Japanese quail (*Coturnix**japonica*) depends on the CS–US interval. J. Exp. Psychol. Anim. Behav. Process..

[6] Boice R, Denny MR (1965). The conditioned licking response in rats as a function of the CS-UCS interval. Psychonom. Sci..

[7] Sutton RS, Barto AG (1992). Reinforcement Learning.

[8] Gerstner W, Lehmann M, Liakoni V, Corneil D, Brea J (2018). Eligibility traces and plasticity on behavioral time scales: Experimental support of neohebbian three-factor learning rules. Front. Neural Circuits.

[9] Roelfsema PR, Holtmaat A (2018). Control of synaptic plasticity in deep cortical networks. Nat. Rev. Neurosci..

[10] Fremaux N, Sprekeler H, Gerstner W (2013). Reinforcement learning using a continuous time actor-critic framework with spiking neurons. PLoS Comput. Biol..

[11] Singh SP, Sutton RS (1996). Reinforcement learning with replacing eligibility traces. Mach. Learn..

[12] Lim DH, Yoon YJ, Her E, Huh S, Jung MW (2020). Active maintenance of eligibility trace in rodent prefrontal cortex. Sci. Rep..

[13] Parker NF (2020). Choice-selective sequences dominate in cortical relative to thalamic inputs to nucleus accumbens, providing a potential substrate for credit assignment. bioRxiv..

[14] Hamid AA, Frank MJ, Moore CI (2021). Wave-like dopamine dynamics as a mechanism for spatiotemporal credit assignment. Cell.

[15] Schultz W, Dayan P, Montague PR (1997). A neural substrate of prediction and reward. Science.

[16] Eshel N (2015). Arithmetic and local circuitry underlying dopamine prediction errors. Nature.

[17] Steinberg EE (2013). A causal link between prediction errors, dopamine neurons and learning. Nat. Neurosci..

[18] Saunders BT, Richard JM, Margolis EB, Janak PH (2018). Dopamine neurons create Pavlovian conditioned stimuli with circuit-defined motivational properties. Nat. Neurosci..

[19] Sharpe MJ (2017). Dopamine transients are sufficient and necessary for acquisition of model-based associations. Nat. Neurosci..

[20] Zhang X (2021). Genetically identified amygdala-striatal circuits for valence-specific behaviors. Nat. Neurosci..

[21] Gallagher M, Graham PW, Holland PC (1990). The amygdala central nucleus and appetitive Pavlovian conditioning: Lesions impair one class of conditioned behavior. J. Neurosci..

[22] Stuber GD (2011). Excitatory transmission from the amygdala to nucleus accumbens facilitates reward seeking. Nature.

[23] Kelley AE, Smith-Roe SL, Holahan MR (1997). Response-reinforcement learning is dependent on *N*-methyl-d-aspartate receptor activation in the nucleus accumbens core. Proc. Natl. Acad. Sci. U.S.A..

[24] Smith-Roe SL, Kelley AE (2000). Coincident activation of NMDA and dopamine D1 receptors within the nucleus accumbens core is required for appetitive instrumental learning. J. Neurosci..

[25] Yagishita S (2014). A critical time window for dopamine actions on the structural plasticity of dendritic spines. Science.

[26] Wieland S (2015). Phasic dopamine modifies sensory-driven output of striatal neurons through synaptic plasticity. J. Neurosci..

[27] Fisher SD (2017). Reinforcement determines the timing dependence of corticostriatal synaptic plasticity in vivo. Nat. Commun..

[28] Urakubo H, Yagishita S, Kasai H, Ishii S (2020). Signaling models for dopamine-dependent temporal contiguity in striatal synaptic plasticity. PLoS Comput. Biol..

[29] Kasai H, Ziv NE, Okazaki H, Yagishita S, Toyoizumi T (2021). Spine dynamics in the brain, mental disorders and artificial neural networks. Nat. Rev. Neurosci..

[30] He K (2015). Distinct eligibility traces for LTP and LTD in cortical synapses. Neuron.

[31] Brzosko Z, Schultz W, Paulsen O (2015). Retroactive modulation of spike timing-dependent plasticity by dopamine. Elife.

[32] Heys JG, Dombeck DA (2018). Evidence for a subcircuit in medial entorhinal cortex representing elapsed time during immobility. Nat. Neurosci..

[33] Sippy T, Lapray D, Crochet S, Petersen CC (2015). Cell-type-specific sensorimotor processing in striatal projection neurons during goal-directed behavior. Neuron.

[34] Otis JM (2017). Prefrontal cortex output circuits guide reward seeking through divergent cue encoding. Nature.

[35] Baldwin AE, Sadeghian K, Kelley AE (2002). Appetitive instrumental learning requires coincident activation of NMDA and dopamine D1 receptors within the medial prefrontal cortex. J. Neurosci..

[36] Murakoshi H (2017). Kinetics of endogenous CaMKII required for synaptic plasticity revealed by optogenetic kinase inhibitor. Neuron.

[37] Iino Y (2020). Dopamine D2 receptors in discrimination learning and spine enlargement. Nature.

[38] Sjostrom PJ, Turrigiano GG, Nelson SB (2001). Rate, timing, and cooperativity jointly determine cortical synaptic plasticity. Neuron.

[39] Abrams TW, Kandel ER (1988). Is contiguity detection in classical-conditioning a system or a cellular property—Learning in aplysia suggests a possible molecular site. Trends Neurosci..

[40] Hawkins RD, Carew TJ, Kandel ER (1986). Effects of interstimulus interval and contingency on classical conditioning of the Aplysia siphon withdrawal reflex. J. Neurosci..

[41] Mariath HA (1985). Operant-conditioning in drosophila-melanogaster wild-type and learning mutants with defects in the cyclic-Amp metabolism. J. Insect Physiol..

[42] Tully T, Quinn WG (1985). Classical conditioning and retention in normal and mutant *Drosophila**melanogaster*. J. Comp. Physiol. A.

[43] Ito I, Ong RC, Raman B, Stopfer M (2008). Sparse odor representation and olfactory learning. Nat. Neurosci..

[44] Demos KE, Heatherton TF, Kelley WM (2012). Individual differences in nucleus accumbens activity to food and sexual images predict weight gain and sexual behavior. J. Neurosci..

[45] Stoeckel LE (2008). Widespread reward-system activation in obese women in response to pictures of high-calorie foods. Neuroimage.

[46] Natsubori A (2017). Ventrolateral striatal medium spiny neurons positively regulate food-incentive, goal-directed behavior independently of D1 and D2 selectivity. J. Neurosci..

[47] Calipari ES (2016). In vivo imaging identifies temporal signature of D1 and D2 medium spiny neurons in cocaine reward. Proc. Natl. Acad. Sci. U.S.A..

[48] Roseberry TK (2016). Cell-type-specific control of brainstem locomotor circuits by basal ganglia. Cell.

[49] Rossi MA (2016). A GABAergic nigrotectal pathway for coordination of drinking behavior. Nat. Neurosci..

[50] Bittner KC, Milstein AD, Grienberger C, Romani S, Magee JC (2017). Behavioral time scale synaptic plasticity underlies CA1 place fields. Science.

[51] Brandalise F, Carta S, Helmchen F, Lisman J, Gerber U (2016). Dendritic NMDA spikes are necessary for timing-dependent associative LTP in CA3 pyramidal cells. Nat. Commun..

[52] Lehmann MP (2019). One-shot learning and behavioral eligibility traces in sequential decision making. Elife.

[53] Pan WX, Schmidt R, Wickens JR, Hyland BI (2005). Dopamine cells respond to predicted events during classical conditioning: Evidence for eligibility traces in the reward-learning network. J. Neurosci..

[54] Amo R, Yamanaka A, Tanaka KF, Uchida N, Watabe-Uchida M (2020). A gradual backward shift of dopamine responses during associative learning. bioRxiv..

[55] Rescorla RA, Holland PC (1982). Behavioral-studies of associative learning in animals. Annu. Rev. Psychol..

[56] Jocham G (2016). Reward-guided learning with and without causal attribution. Neuron.

[57] Hikida T, Kimura K, Wada N, Funabiki K, Nakanishi S (2010). Distinct roles of synaptic transmission in direct and indirect striatal pathways to reward and aversive behavior. Neuron.

[58] Suzuki E, Nakayama M (2011). VCre/VloxP and SCre/SloxP: New site-specific recombination systems for genome engineering. Nucleic Acids Res..

[59] Grieger JC, Choi VW, Samulski RJ (2006). Production and characterization of adeno-associated viral vectors. Nat. Protoc..

