# Supplementary material for: A behavioural correlate of the synaptic eligibility trace in the nucleus accumbens
Source: Sci Rep. 2022 Feb 4;12:1921. doi: 10.1038/s41598-022-05637-6 (PMC8817024; doi:10.1038/s41598-022-05637-6)
Supplement: Supplementary file 1 — Supplementary Figures. [file 41598_2022_5637_MOESM1_ESM.pdf]

A behavioural correlate of the synaptic eligibility trace in the nucleus accumbens

Kenji Yamaguchi[1,2], Yoshitomo Maeda[1,2], Takeshi Sawada[1,2], Yusuke Iino[1,2], Mio Tajiri[1,2], Ryosuke Nakazato[1,2], Shin Ishii[2, 3], Haruo Kasai[1,2], and Sho Yagishita[1,2]

[1]Laboratory of Structural Physiology, Center for Disease Biology and Integrative Medicine, Faculty of Medicine, [2]International Research Center for Neurointelligence (WPI-IRCN), UTIAS, The University of Tokyo, Bunkyo-ku, Tokyo, and [3]Graduate School of Informatics, Kyoto University, Yoshida-Honmachi, Kyoto, Japan

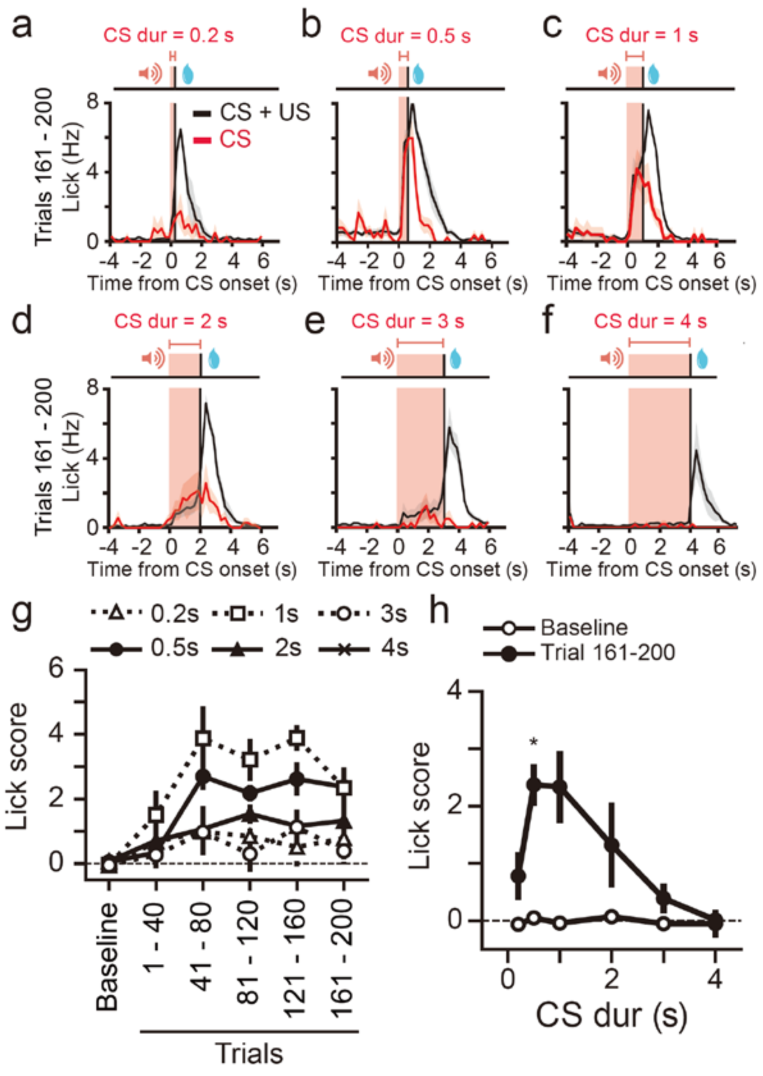

Figure S1. Conditioning with various CS durations.

a–**f**) Averaged PSTHs of the licking responses with CS duration of 0.2 s (a, *n* = 4 mice), 0.5 s (b, n = 7 mice), 1 s (c, *n* = 5 mice), 2 s (d, *n* = 7 mice), 3 s (e, *n* = 4 mice), or 4 s (f, n = 5 mice) during trials 161–200. The plot in (b) is the same as that at the bottom of Fig. 1f and Fig. 2d. Red shades indicate the period of CS presentation.

g) The lick scores for each condition are plotted against time. The averaged lick scores in the CS-only trials were plotted against time. Each symbol represents the CS duration.

h) The effect of CS duration on conditioning. The averaged lick scores in the CS-only trials included during conditioning trials 161–200 were plotted against CS durations. Wilcoxon signed-rank test. * $P < 0.05$.

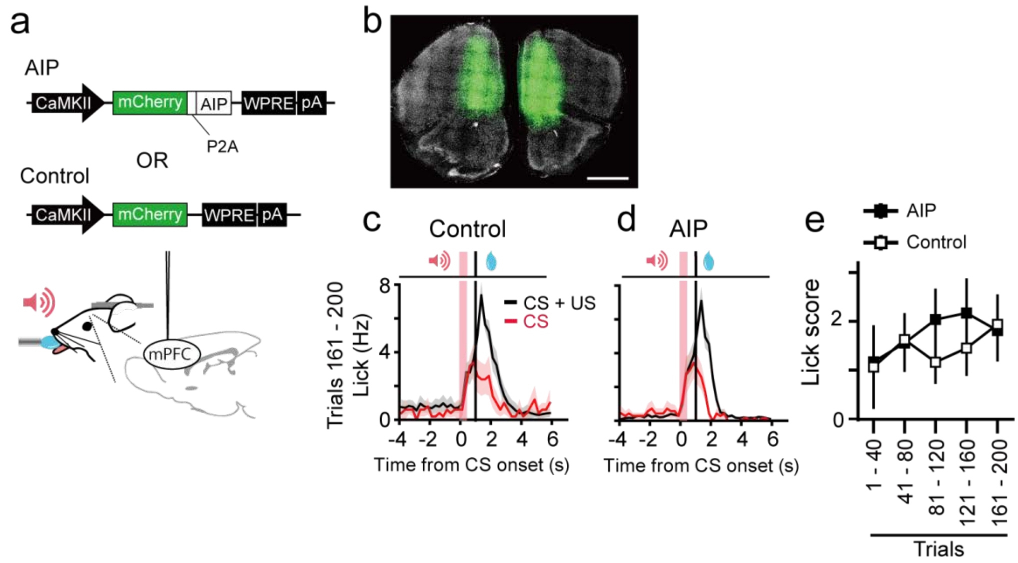

Figure S2. The effect of a CaMKII inhibitory peptide (AIP) on the mPFC on conditioning.
a) Viral constructs and schematics of the injection.
b) Confocal images of mCherry fluorescence (green) and DAPI (white) from a coronal slice, including the PFC. The scale bar indicates 1 mm.
c, d) Averaged PSTHs of the licking responses in CS + US (black) and CS (red) conditionings from mice injected with control (d, n = 5 mice) or AIP (c, n = 7 mice).
e) The peak lick scores plotted against time for the conditions indicated.

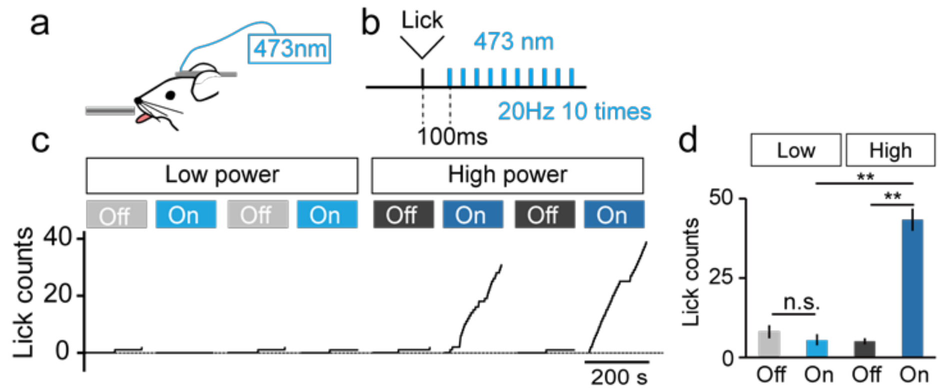

Figure S3. Operant conditioning with stimulation of the axon terminals from the BLA to the NAc.

a, b) Operant conditioning using stimulation of the axon terminals from the BLA. The lick port detected licking to trigger blue laser stimulation (20 Hz, ten times, 5 ms pulse width) with a delay of 100 ms (b). No water was given during this operant protocol. Laser power was adjusted to 1~3 mW for a low power condition and 5~15 mW for high power condition.

c) Representative traces of cumulative licking counts during low power (2.5 mW) and high power (5 mW) conditioning. Conditions with laser-off and on were alternated.

d) Averaged total lick counts for the condition indicated. Kruskal–Wallis test ** $P < 0.01$.

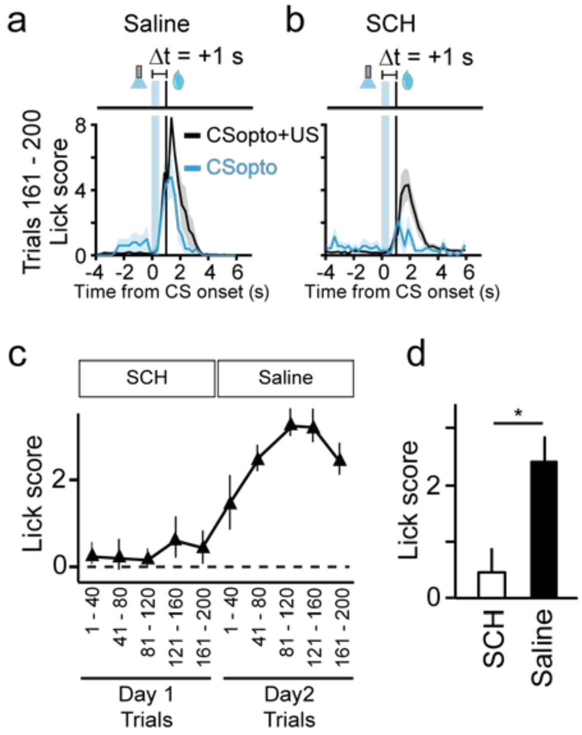

Figure S4. Effects of a dopamine D1 receptor blocker on Pavlovian conditioning with CSopto.
a, b) Averaged PSTHs of the licking responses during the last 20% of the trials (161–200, bottom) for a dopamine D1 receptor blocker (SCH23390, 0.25 or 0.5 mg/kg, i.p) (a) or saline (b). On day 1, mice received intraperitoneal injections of SCH23390 before conditioning with CSopto with a delay of +1 s as in Fig. 5. The next day, the mice were administered saline, and the conditioning was repeated. $n = 5$.
c) Lick scores plotted against time.
d) The effect of a dopamine D1 receptor blocker. Averaged lick scores from 4 CS-only trials during trials 161–200 were plotted for each condition. * $P < 0.05$, two-sided Mann–Whitney U test, $n = 5$ mice.

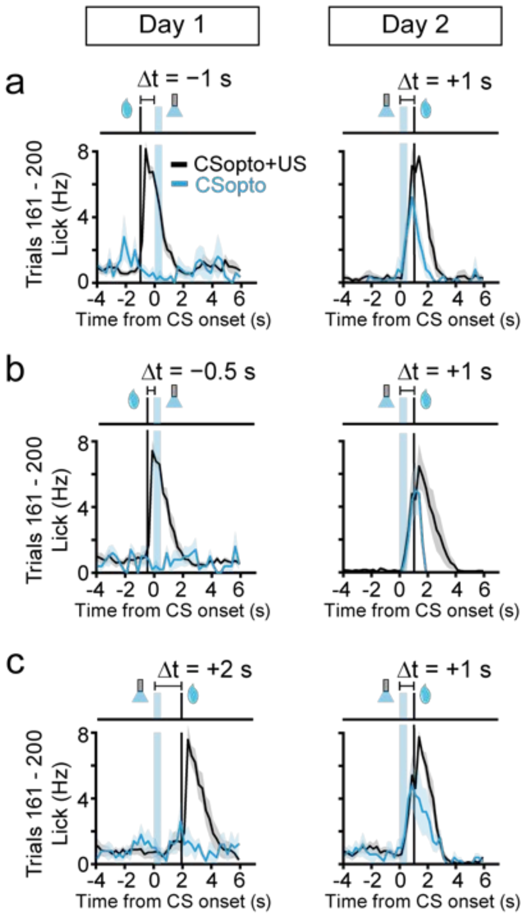

Figure S5. Positive controls for conditioning with CSopto.
a, b, c) Averaged PSTHs of the licking responses during the last 20% of the trials (161–200, bottom). Mice allocated to Δt = −1 s (a, *n* = 5 mice, left), Δt = −0.5 s (b, *n* = 5 mice, left), and Δt = 2 s (c, *n* = 5 mice, left) on day 1 were conditioned with Δt = +1 s on day 2 (right).
